# A Pilot Study of Junior Medical Officers in Sydney, Australia: Knowledge and Attitudes Towards Artificial Intelligence

**DOI:** 10.7759/cureus.117269

**Published:** 2026-09-30

**Authors:** John W Coen, Stephen D McCarthy, Helen C Anderson

**Affiliations:** 1 Hospital Medicine, Northern Sydney Local Health District, Sydney, AUS

**Keywords:** artificial intelligence, artificial intelligence (ai) in healthcare, healthcare technology, health professional's education, junior doctor, public healthcare

## Abstract

Introduction

Artificial intelligence (AI) use is growing generally and specifically in healthcare. It has multiple applications across medicine, including summarising patient consultations, interpreting investigations and improving patient flow. Junior Medical Officers (JMOs) working in Northern Sydney Local Health District (NSLHD), in New South Wales (NSW), are amongst the first generations of clinicians to experience the broadest expansion of the use of AI and are likely to determine the guidelines for its use. However, they remain an underrepresented group in the literature.

Aim

This study assesses and evaluates the knowledge and attitudes of JMOs towards AI, thoughts on applications of AI and willingness to incorporate it into future practice.

Methods

An online survey was sent to JMOs in two hospitals in the Northern Sydney Local Health District in Sydney, Australia. Responses were analysed qualitatively and quantitatively.

Results

Of the JMOs surveyed, 37.5% rated their knowledge of AI generally as good or very good, 33.3% rated it as average and 29.2% rated it as poor. Knowledge of AI in healthcare was self-reported as good by 23.3%, average by 33.3%, poor by 36.7% and very poor by 6.7% of respondents. When responding to questions about frequency of use of AI in clinical work, 10% of JMOs responded that they use AI daily, 30% weekly, 3% fortnightly and 23% monthly, with 34% never using AI. JMOs expressed broadly positive attitudes towards AI, with 10.3% viewing it very positively, 55.3% positively, 10.3% negatively and 3.4% very negatively, with 20.7% holding neutral views.

JMOs felt the main advantages of AI were “freeing time from documentation”, “processing large amounts of data” and “searching through data for best practice”. The main concern identified was the output of incorrect information by AI.

Respondents felt the future roles for AI were scribing and summarising notes, real-time translation, medical education and interpreting medical imaging. Amongst those surveyed, 76% were open to using AI in the future, and 83% said they would be more likely to use AI if the programme was endorsed by the local health district.

JMO responses indicated that they felt that AI should be included in medical education curricula, with 84% responding in the affirmative.

Conclusion

This study found that JMOs in Northern Sydney Local Health District have varying levels of understanding of AI but broadly view the technology positively. Although current use of AI is relatively limited, participants identified significant potential for AI to reduce administrative workload, particularly through clinical documentation tasks such as scribing and summarising patient notes.

Many respondents indicated they would be more likely to use AI in both clinical and non-clinical settings if it were formally endorsed by the local health district. Participants also expressed a strong interest in further AI education and supported its inclusion in medical training. Human oversight was consistently regarded as essential.

The current lack of clarity remains a key barrier to implementation. As AI technologies continue to evolve, healthcare organisations, policymakers, and clinicians, namely, JMOs, share responsibility for ensuring their safe, ethical, and effective integration into practice.

## Introduction

Artificial intelligence (AI), a concept initially originating from the work of Alan Turing in the mid-20th century, is defined as a machine or computer simulating the intelligence of a human [1]. Khan et al. [2] list its common functionalities at present as including the generation of intelligible prose, the summary and simplification of large bodies of literature (often scientific), statistical analysis and the generation and analysis of images. Frequently used programmes worldwide are ChatGPT and Google’s Gemini, although AI is also used in certain software created by Meta and in X’s Grok. Usership has increased greatly over the last 12-24 months, with an estimated 22% of people using ChatGPT on a weekly basis, increasing to 60% amongst 18-25-year-olds [3].

In medicine more specifically, AI has been proposed to have applications for improving patient flow and triage accuracy, interpreting investigations, summarising patient consultations and reviews and determining the need for follow-up appointments [4]. More than simple proposals, however, the actual, and sanctioned, use of AI is rapidly growing in the healthcare field; it has been deployed in specialties where there are large amounts of data to analyse, including pathology and radiology [5].

In the Australian state of New South Wales (NSW), a Junior Medical Officer (JMO) is a fully qualified medical practitioner who is still in training and has not yet applied for a specialist or General Practitioner post [6]. They are referred to as “resident doctors” in some other countries. This subset of doctors was not widely exposed to AI through their medical studies and early careers; however, they will likely experience the period of most significant AI expansion into medical practice. As such, they may be called upon to make decisions regarding its regulation and implementation. Given the typical ages of JMOs, they have grown up with evolving technology and therefore are (broadly speaking) technologically literate; thus, they may have a unique understanding of AI as a new technology compared to more senior colleagues, or other decision-makers.

The majority of research into medical practitioner attitudes towards AI comes from Europe and the United States and focuses, with very good reason, on experienced specialists and consultants [7-11]. As such, this paper aims to summarise current views on AI amongst JMOs in NSW, an underrepresented cohort on account not only of their geographical location but also of their relatively low position in the traditional medical hierarchy, and to evaluate attitudes on future applications of AI and willingness to incorporate AI into future practice.

## Materials and methods

An electronic survey, produced on Microsoft Forms (Microsoft Corp., Redmond, WA), was sent to JMOs working at two separate hospitals, Mona Vale Hospital and Hornsby Ku-ring-gai Hospital, in the Northern Sydney Local Health District (NSLHD), part of the NSW public health system. A link to the survey was sent to them via their official NSW Health email address, and reminders were sent via the WhatsApp messaging platform. Participation was voluntary, and all responses were anonymous. The questionnaire was left open for four weeks, and reminder messages and emails were sent at one and three weeks. Thirty responses were received in total. The questionnaire was distributed initially to approximately 200 JMOs due to inaccurate mailing lists; of those, only 86 were eligible to complete the survey, giving a response rate of 34.9%. This provided a 14.5% margin of error at 95% confidence; however, this was felt to be acceptable given the exploratory nature of the study.

The survey, shown in the Appendices, consisted of questions on demographics, experience and comfort utilising AI, and perceptions of current and future roles of AI in medicine. These questions were based on the findings of a detailed review of current literature relating to AI in medicine in other healthcare systems, clinician concerns regarding AI and clinician considerations of how AI may be used in their future practice. The questions were reviewed by a small number of colleagues to ensure they were relevant and clear.

Inclusion criteria included the following: currently working as a JMO and working in a public hospital of the Northern Sydney Local Health District (NSLHD), either Mona Vale Hospital or Hornsby Ku-Ring-Gai Hospital. Exclusion criteria were as follows: working in a specialist role or as a qualified General Practitioner (GP), working at a different public hospital in NSLHD or working in a private hospital.

All those who completed the questionnaire satisfied the inclusion criteria.

The majority of questions in the survey were in a multiple-choice format to aid with ease of answering, whilst a small number of “yes/no” and Likert scale questions were also included. This style and mix of questions were chosen to gather detailed information on the relatively complex concept of AI and its subtopics related to medicine. When answering in the negative, respondents were prompted with a follow-up question to explain their stance if they wished. Many of the multiple-choice questions had an “other” option for participants to use, should they have wished to provide more detail.

The Clinical Governance and Quality Improvement teams for the Local Health District were contacted, and the study proceeded with no need for formal ethical approval given that it involved anonymous, unidentifiable data collected from clinicians alone; no patient data was collected. This was reviewed by the Director of Medical Services at Hornsby Hospital. Raw data for both qualitative and quantitative answers were not shared outside the research team. Analysis was done using Microsoft Excel, and longer answers underwent thematic analysis.

## Results

Demographics

The demographic characteristics of the respondents are shown in Table 1.

**Table 1:** Age and years of experience of the respondents Note: One respondent did not fill in their age.

Characteristics	Number of respondents
Age	
25-29 years	20
30-34 years	4
35-39 years	2
≥40 years	3
Years of experience	
<2 years	11
2-5 years	13
5-10 years	4
>10 years	2

Knowledge and use of AI

Knowledge of AI was evenly distributed amongst the four self-assessment categories. Over one-third of the participants indicated that they have a good or very good subjective knowledge of AI, representing 37.5% of the study cohort. A similarly sized group felt they had an average level of knowledge (33.3%). The remaining 29.2% felt they had poor knowledge of AI. These proportions remained similar when asked about knowledge of AI in healthcare (6.7% very poor, 36.7% poor, 33.3% average and 23.3% good). There was no correlation between either years of experience or age and knowledge of AI generally or in healthcare.

When asked about use of AI in their clinical work, 10% used it daily, 30% weekly, 3% fortnightly and 23% monthly, whilst the remaining 34% stated that they never use AI in their clinical work. Regarding personal education, 37% used AI daily, 23% weekly, 10% fortnightly and 17% monthly, with the remaining 13% stating that they never used AI for their education.

Attitudes towards AI

The JMOs surveyed broadly responded that they viewed AI positively. Of the respondents, 10.3% said that they viewed AI very positively, 55.3% positively, 10.3% negatively and 3.4% very negatively, with 20.7% having neutral views. Most respondents (55%) felt that AI would impact their clinical practice in a positive manner, whilst 7% felt that the impact would be very positive. Moreover, 7% felt that the impact would be negative, and 3% very negative. Additionally, 28% felt that any impact would be neutral.

JMOs were asked to select what they felt were the four main advantages of AI, from a list, with an option for “other” advantages. The most reported advantages were “freeing time from documentation”, selected 26 times; “processing large amounts of data” (with respect to formulating discharge letters and patient summaries), selected 22 times; and “searching through data for best practice”, selected 15 times. The results for this question are shown in Figure 1.

**Figure 1:**
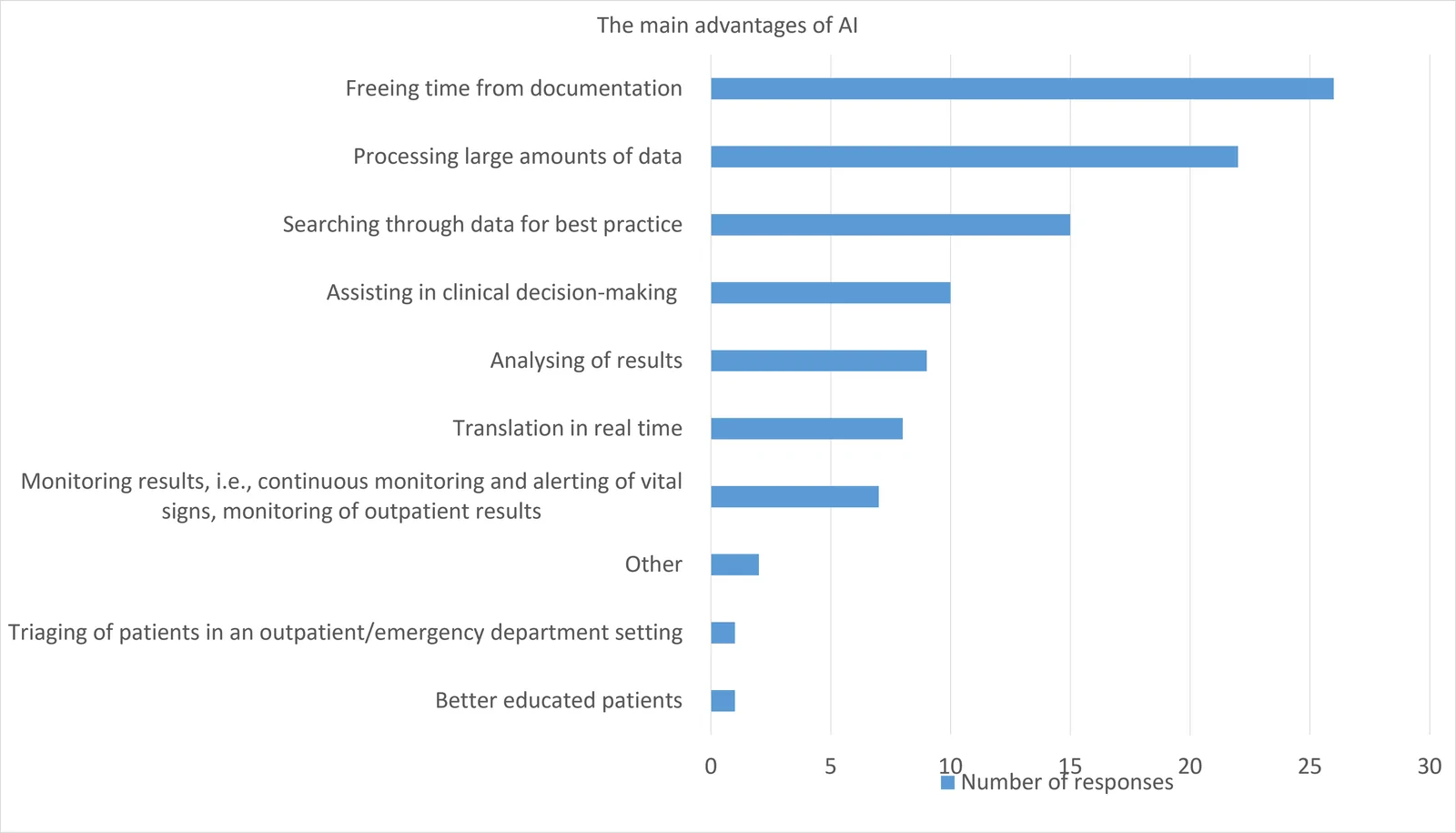
Main advantages of AI AI: artificial intelligence

When asked about the main concerns surrounding AI at present, JMOs most commonly cited the output of incorrect information (24 respondents), issues with confidentiality (19 respondents) and that its use may lead to overreliance on AI (14 respondents). Only 4 respondents identified the eradication of their job as a concern. The results are shown in Figure 2.

**Figure 2:**
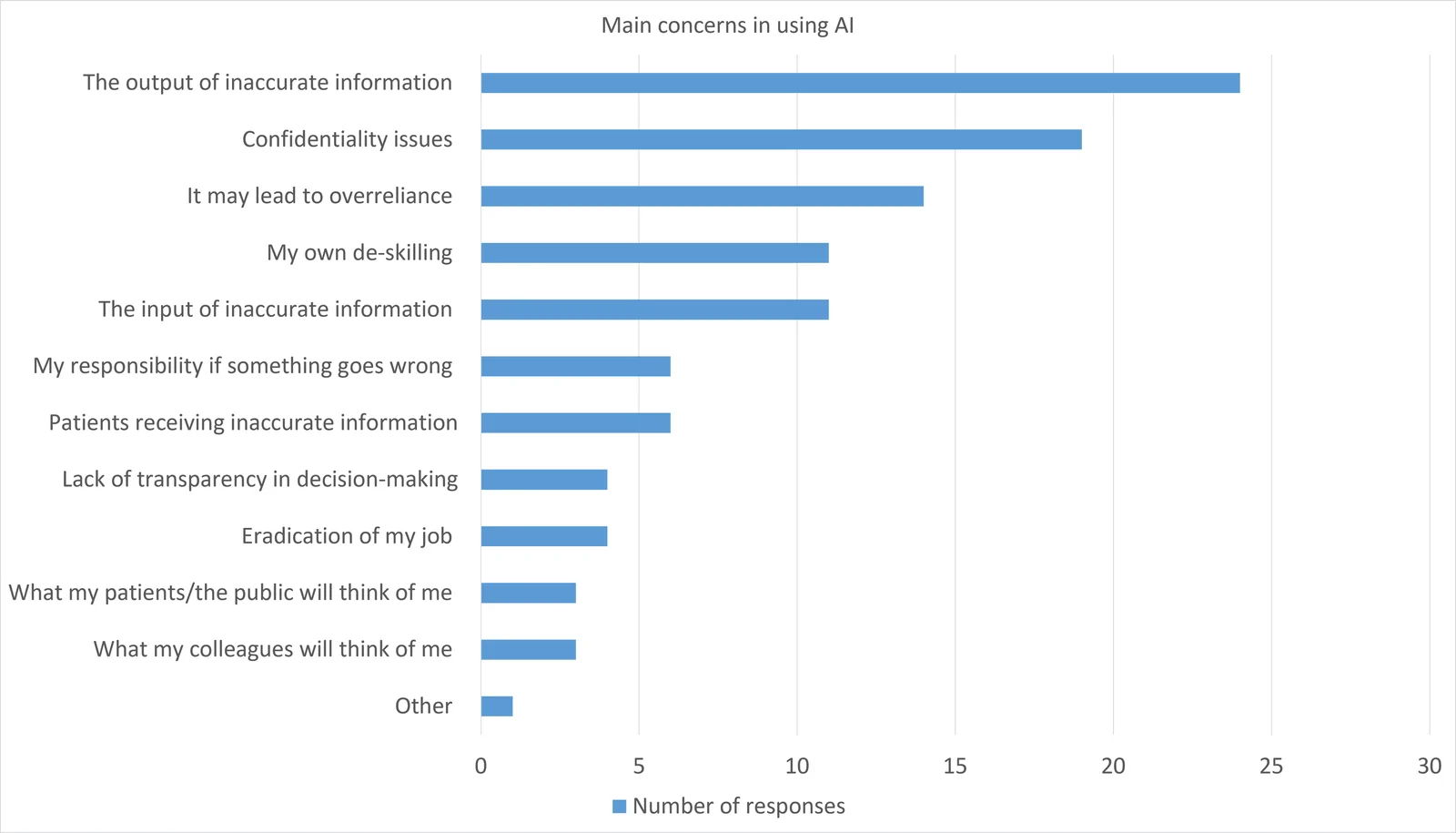
Main concerns in using AI AI: artificial intelligence

JMOs were also asked specifically if, at present, they would be comfortable acting on a medical imaging report produced by AI; 20% responded “yes”, and 80% responded “no”. The results are shown in Figure 3.

**Figure 3:**
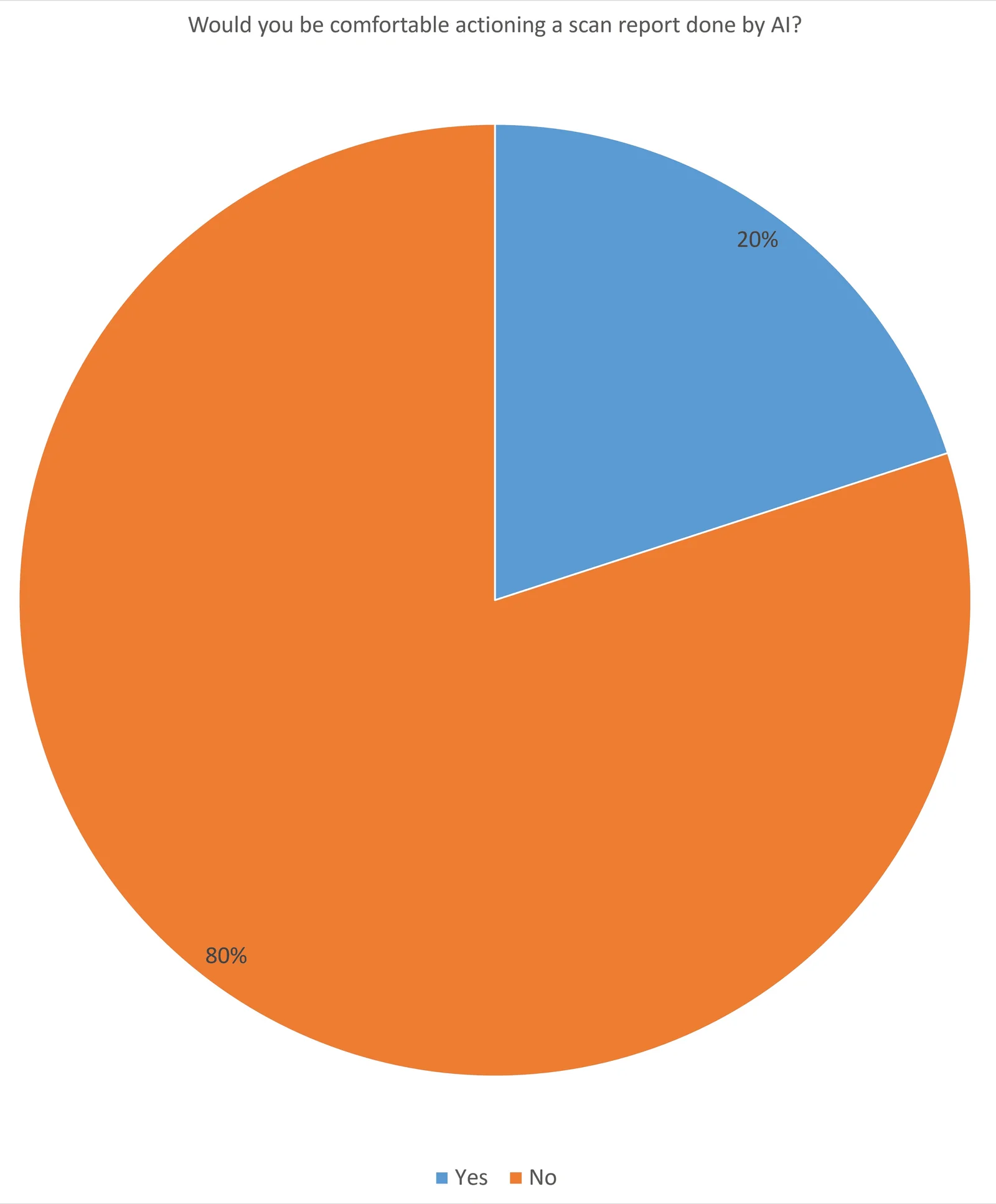
Comfortability in actioning a scan report written by AI AI: artificial intelligence

Thematic analysis of reasons for a “no” response to questions around AI-generated imaging reports was varied. The most common reasons given mentioned a preference for the scan to be reviewed by a human first (either the respondent themselves or a radiologist) and concerns over the accuracy of an AI interpretation at this stage of development. Verbatim responses are shown in Table 2.

**Table 2:** Verbatim responses qualifying reasoning for negative responses towards actioning scan reports done by AI, with associated demographics of the respondent AI: artificial intelligence

Respondent demographics	Response
29 years old, 2-5 years’ experience	“I would be unsure of its accuracy, much more comfortable actioning from a radiologist”.
31 years old, 2-5 years’ experience	“I would like a radiologist to verify the scan at a minimum”.
Age not provided, 5-10 years’ experience	“Algorithms do not always detect subtle variances which clinical experience does”.
26 years old, <2 years’ experience	“Depending on the scan, I don’t think AI strictly outperforms human interpretation”.
42 years old, 5-10 years’ experience	“Too early; have not seen evidence of accuracy yet”.

When asked whether they would be comfortable acting on a summary of blood tests produced by AI, 43% responded “yes” and 57% “no”. These results are shown in Figure 4.

**Figure 4:**
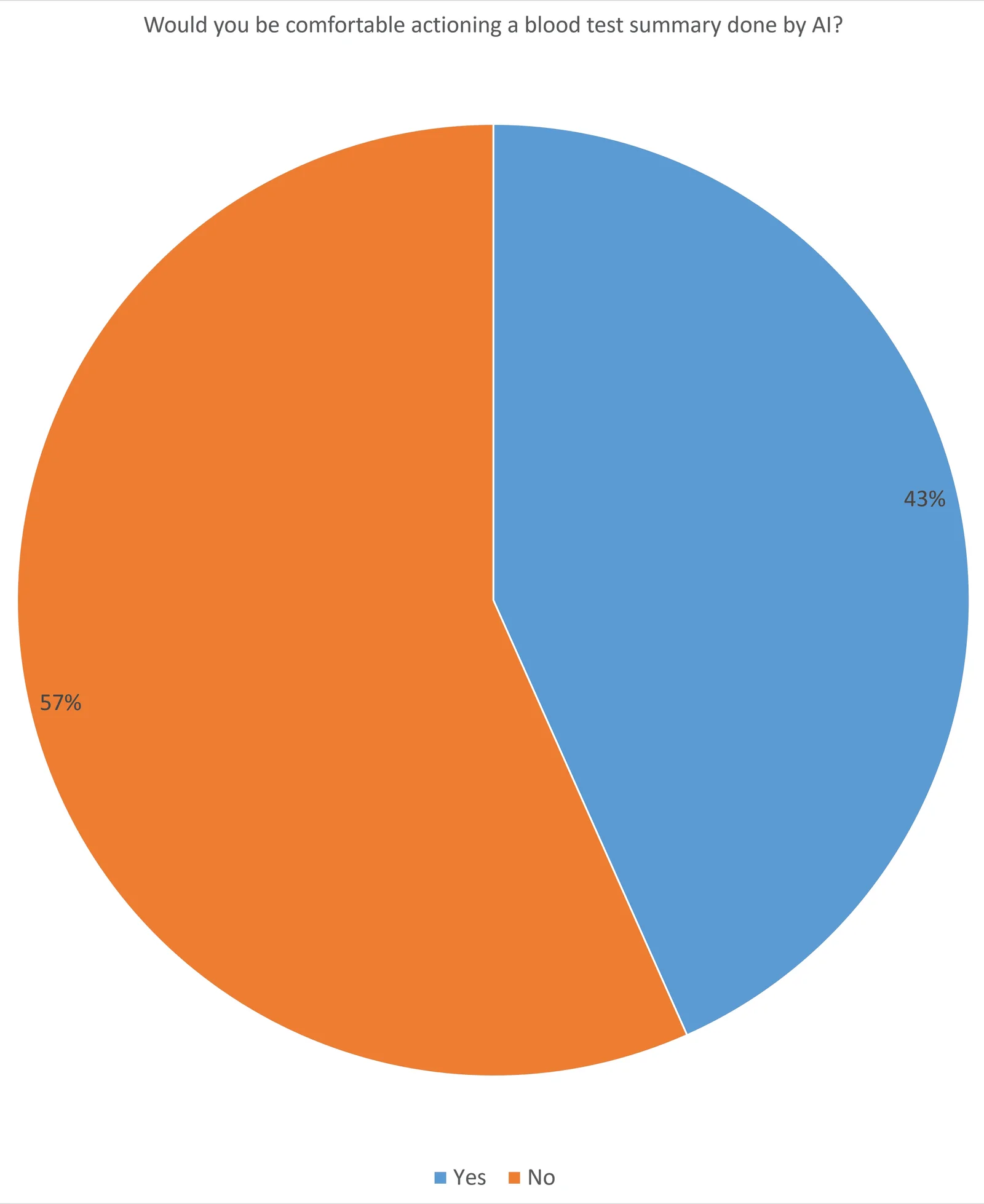
Comfortability in actioning a blood test summary produced by AI AI: artificial intelligence

Thematic analysis of reasons for a “no” response revealed concerns over the accuracy of AI and the inability of AI to correlate results to the clinical context as the main concerns. Verbatim responses are shown in Table 3.

**Table 3:** Verbatim responses qualifying reasoning for negative responses towards actioning blood test reports done by AI, with associated demographics of the respondent AI: artificial intelligence

Respondent demographics	Response
29 years old, 2-5 years’ experience	“The total clinical picture may not be taken into account by the AI”.
26 years old, <2 years’ experience	“Not confident in the reliability and accuracy of AI”.
26 years old, <2 years’ experience	“I don’t know whether it is reliable”.

Regarding whether they would be comfortable with AI scribing and summarising a patient consultation or not, 88% of JMOs responded in the affirmative, whilst 12% responded negatively.

A large majority of respondents felt that clinicians should disclose the use of AI to their patients: 89% of JMOs replied yes, whilst 11% replied no. With regard to the question of responsibility in an adverse event where AI was used, 38% responded that it was solely the clinician’s responsibility, 24% responded that it was the shared responsibility of the clinician and local health district/hospital, 21% responded that it was the shared responsibility of the clinician and the AI developer, 14% responded that it was the shared responsibility of the local health district/hospital and the AI developer and 3% stated that it was a combination of the clinician, AI developer and the local health district/hospital. No respondents felt that it was the sole responsibility of the AI developer. The results are shown in Figure 5.

**Figure 5:**
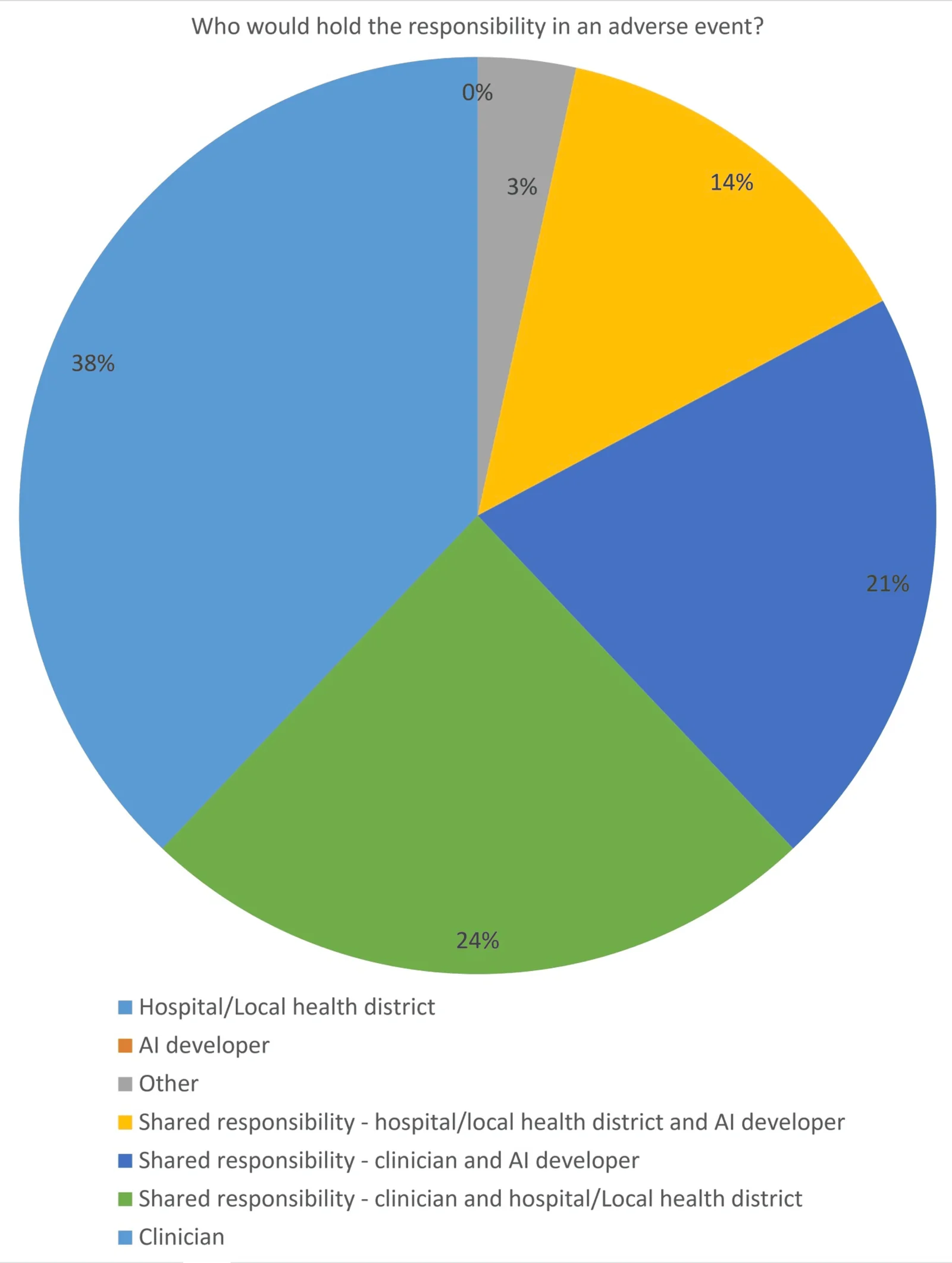
Responsibility in an adverse event where AI was used AI: artificial intelligence

Future uses of AI

When JMOs were asked to identify the main future roles of AI in medicine, the most common responses were scribing notes (25 responses), summarising notes (23 responses), real-time translation (16 responses), medical education (18 responses), interpreting scans (16 responses) and prognostication/predicting disease trends (14 responses). The results of this question are shown in Figure 6.

**Figure 6:**
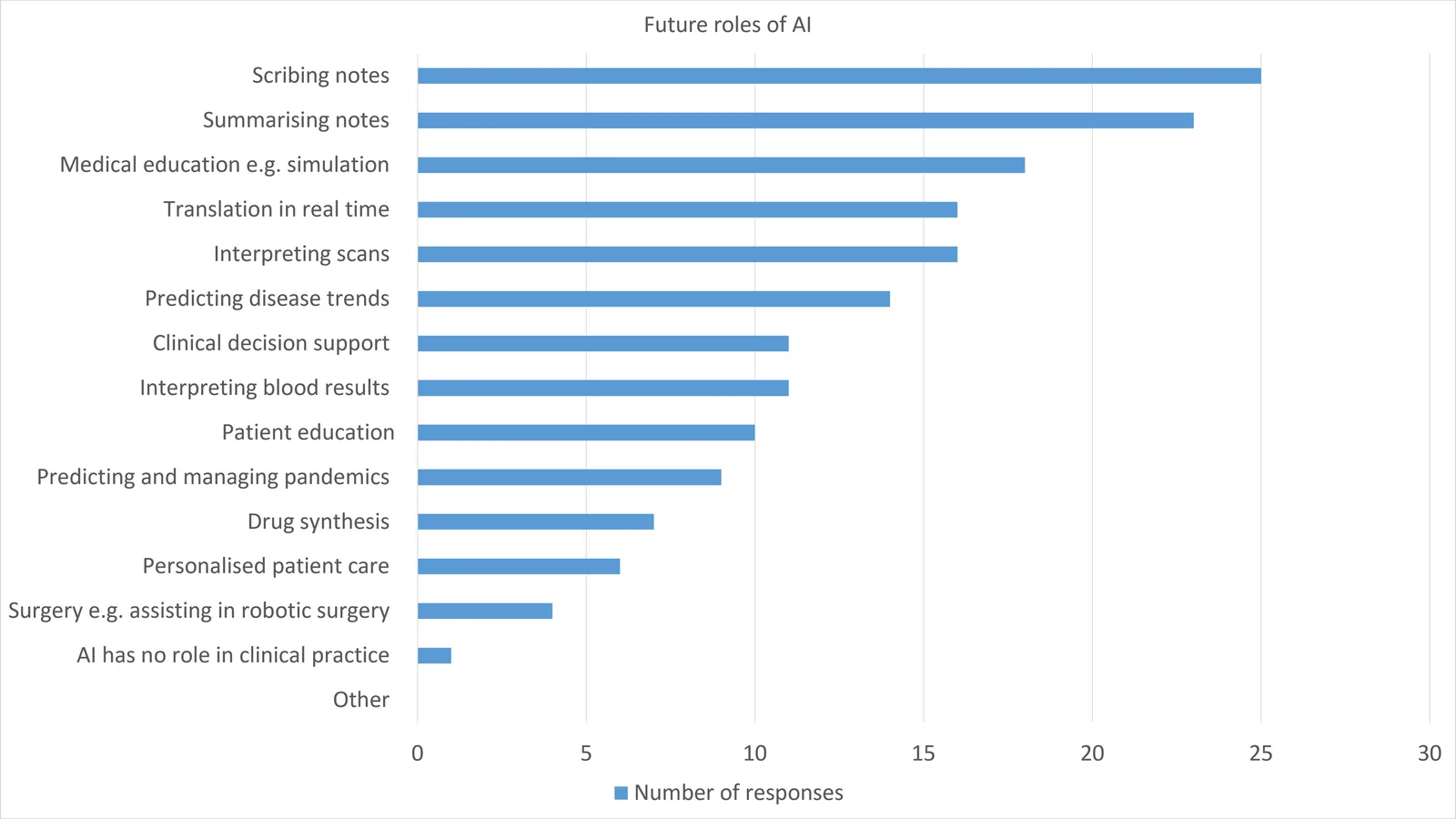
Future roles of AI in healthcare AI: artificial intelligence

Of the respondents, 79% stated that they would be open to incorporating AI into their clinical practice in the future, 7% replied that they would not be open to incorporating AI into their clinical practice in the future, whilst the remaining 14% were unsure. These results are shown in Figure 7.

**Figure 7:**
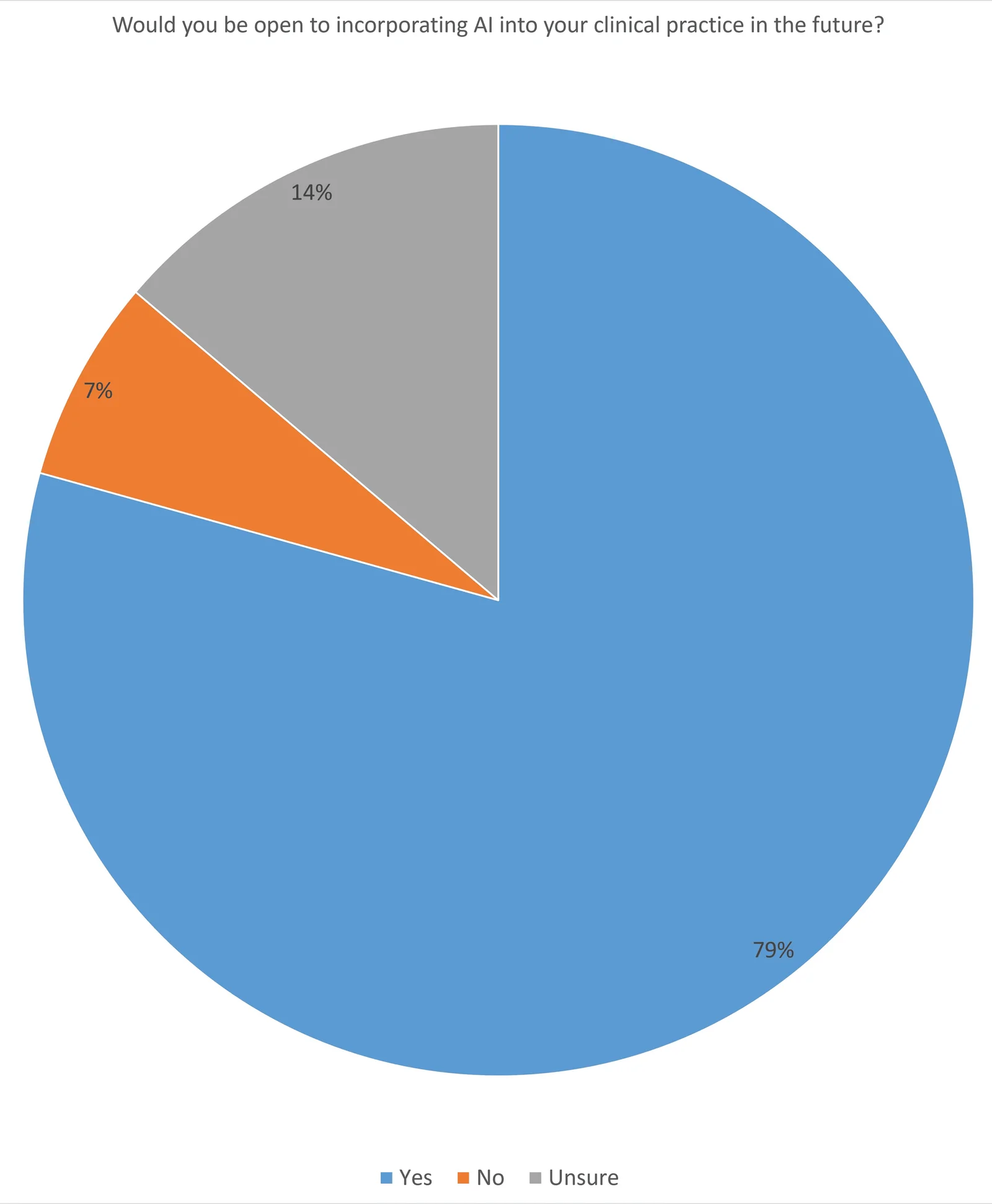
Openness to incorporating AI into clinical practice in the future AI: artificial intelligence

JMOs were also asked about incorporating AI into other areas of their roles outside of clinical practice; 90% responded “yes”, 7% responded “no” and 3% responded “unsure” (Figure 8).

**Figure 8:**
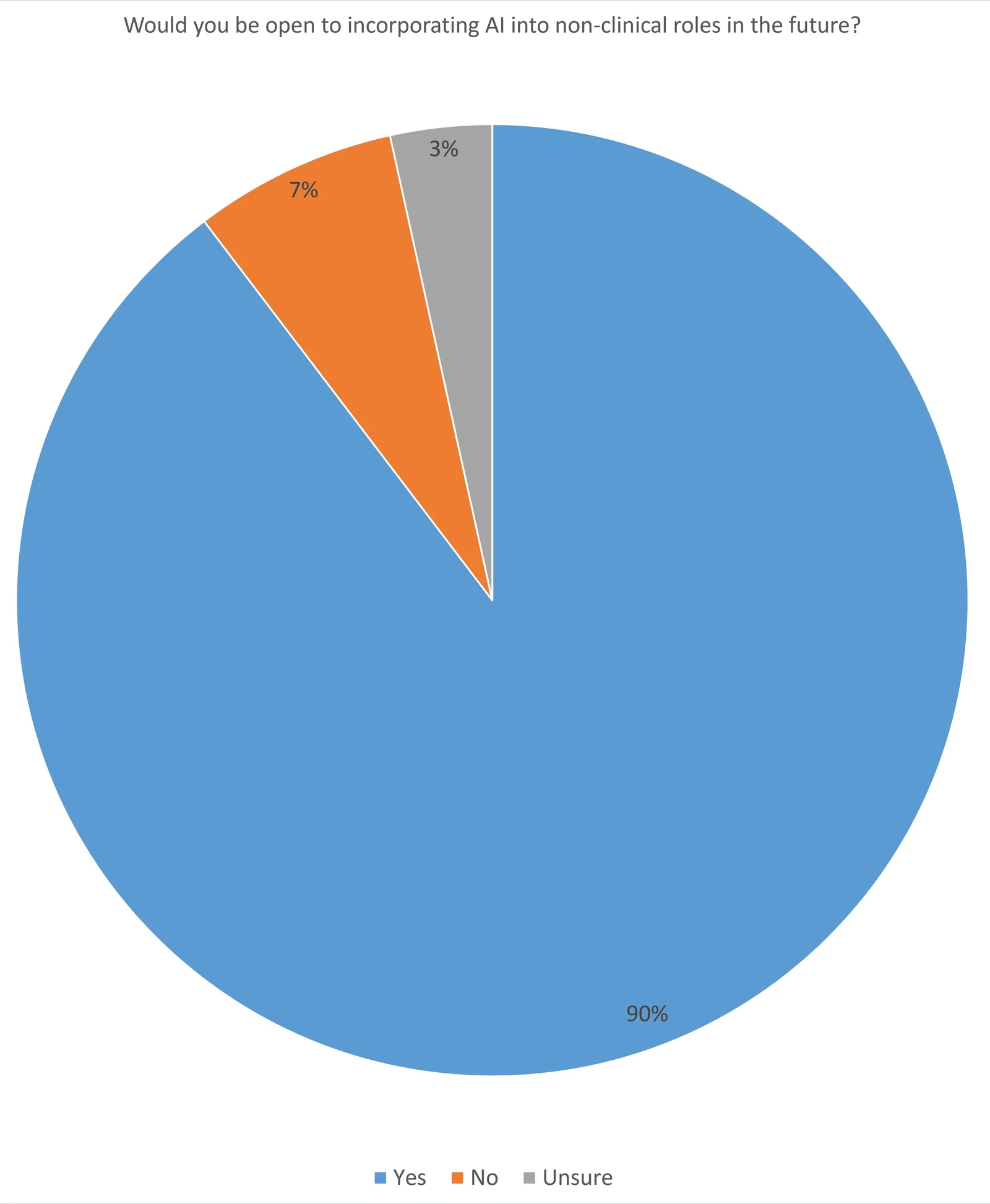
Openness to incorporating AI into non-clinical work in the future AI: artificial intelligence

When asked specifically about whether they would be more likely to incorporate an AI programme if it were endorsed by their local health district, 78% of the JMOs responded “yes”, 11% responded “no” and 11% responded “unsure”. These results are shown in Figure 9.

**Figure 9:**
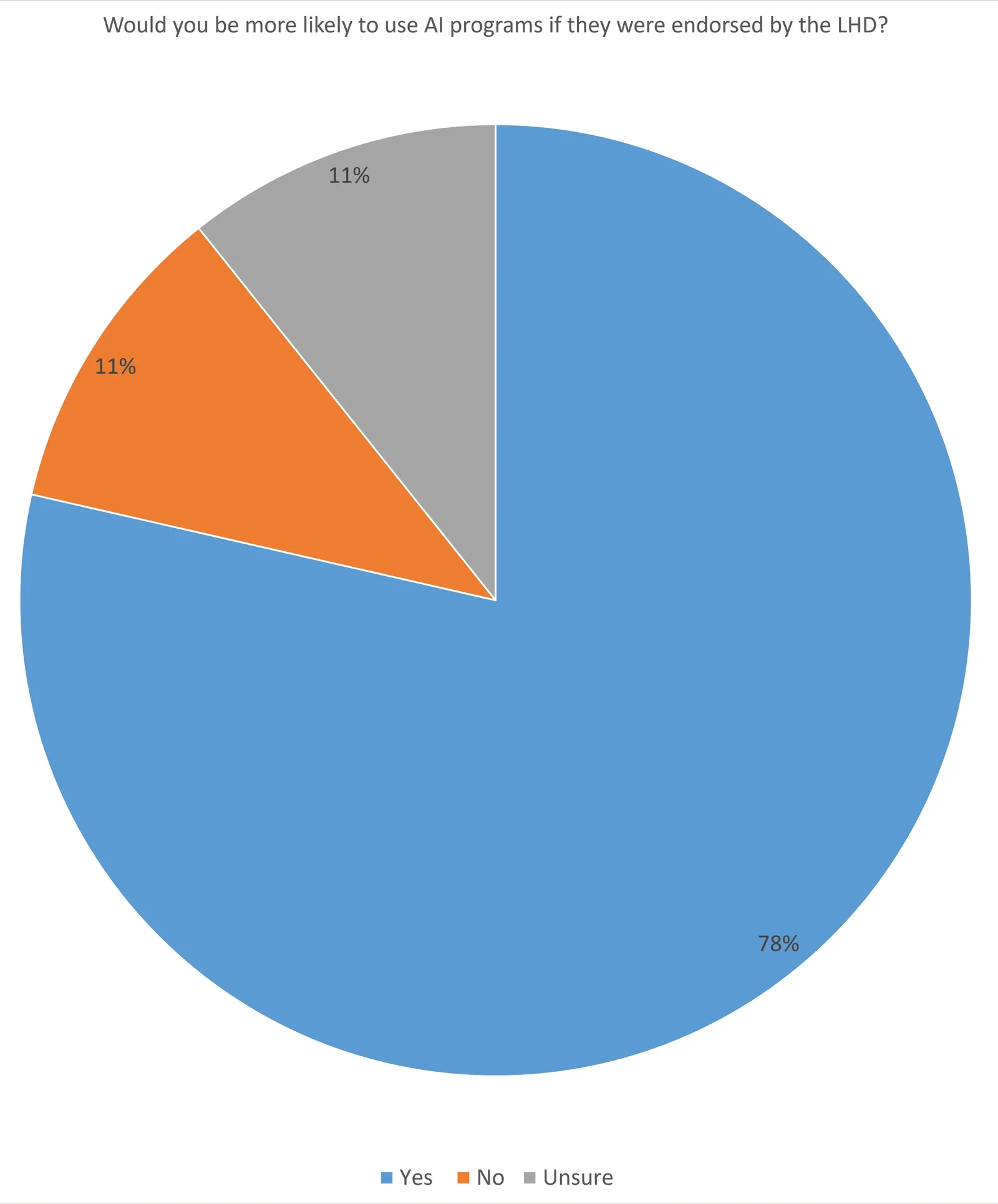
Likelihood of AI use if a programme was endorsed by the local health district AI: artificial intelligence

JMOs were asked in what areas they would like to see AI implementation focused, and these responses underwent thematic analysis. The main areas that respondents identified were those most commonly cited above: scribing and documentation, and summarising patient data. A vast majority of JMOs felt that an introductory knowledge of and approach to AI should be included in medical education curricula: 79% responded “yes”, 7% responded “no” and 14% responded “unsure”.

## Discussion

The (international) landscape

The introduction of new technologies into any professional field generally garners some degree of debate and controversy; thus, it is helpful to gain a sense of the state of play in other countries with regard to technologies used by JMO equivalents. Smartphone use, for instance, was reported to be increasingly prevalent amongst junior doctors in the UK some 10 years ago, and comfort with this technology allowed for its seamless integration into the workplace, for use with medical applications and professional communication [12]. In the intervening decade, they have become an invaluable part of the junior doctor’s toolbox internationally across the world. A synthesis of qualitative evidence by Glenton et al. [13] listed the functions of smartphones as including two-way communication with nursing staff, relaying of patient information (e.g., imaging) to more senior colleagues for advice, storage of images for use in teaching scenarios and sources of information regarding pathology or medication. They also warn of the risks that are already embedded in workplaces where smartphones are commonplace. These expectations include being easily contactable even outside of working hours, use of doctors’ own internet data, breaches in confidentiality with the storage of patient information on personal devices and the widening of gaps between better-resourced and less well-resourced settings, where smartphones may not be as available to doctors.

The relationship doctors have with AI, whilst in its infancy, may yet be tracing a comparable arc. In this study, most JMOs reported using AI fortnightly or less frequently, with approximately one-third of participants reporting never having used it in a clinical context. It is interesting to compare these figures with the reported frequency of AI use amongst doctors globally in their day-to-day work. Alkhatieb and Subke [14] reported in their study of Saudi Arabian physicians that 70% of participants had never used AI in their clinical work. When compared to a survey from the American Medical Association (AMA) from March 2026, up to 80% of doctors reported using AI in their clinical practice. The same survey showed that only 19% of the AMA respondents reported never having used AI in a clinical context, a decrease from 34% in 2023 [15]. This latter figure closely mirrors the results of this study, suggesting that use in Australia could increase in a similar fashion over the next number of years.

In some cases, though, AI is already being utilised internationally to reported good effect. The implementation of an AI that can predict admissions and discharges based on the electronic patient record has already been undertaken in Canada, reportedly to good effect [16]. Australian uses are discussed in further detail below.

Local endorsement and regulation

For a majority of respondents in this survey, it appears that one of the main barriers to using AI is that there is no programme specifically endorsed by the NSLHD. These concerns are not unique to Sydney, or even Australia. A study of Brazilian doctors found that a similar proportion of those surveyed felt that AI programmes should be regulated by a governmental agency [17], whilst qualitative data from Sweden (whose model of public healthcare is similar to those found in Australia) revealed similar barriers to the wider implementation of AI. This Swedish survey explicitly identified the need for government support to develop and integrate AI into the health system [18].

The literature suggests a wide range of future uses for AI, over a range of timeframes, and the summary of patient notes was put forth as a main future use of AI by JMOs in this study. Buch et al. [4] suggest a particular relevance in general practice, where large amounts of data from a patient with a chronic condition could be summarised by an AI programme and presented to a clinician prior to their appointment, to avoid the clinician needing to read through a large volume of medical notes. A system like this would seem to be favourable with the JMOs surveyed in this study. However, NSW Health, the governmental department responsible for healthcare in NSW, produced an Information Bulletin in 2024 discussing the uses of AI; it lists using AI to summarise a report using data that is not publicly available (such as personal health data) as an inappropriate use of the technology, due to the confidential nature of a person’s health data [19].

Outside of the barrier in this salient concern of confidentiality, the broader question of regulating AI in a healthcare setting in NSW, or Australia more generally, regardless of purpose, is grey. Guidance from the Therapeutic Goods Administration (TGA), Australia’s national regulatory body for medications, states that an AI scribe does not need to be regulated as a medical device under the TGA if it does not diagnose, investigate or provide any sort of medical information back to the user [20]. Therefore, a programme such as Heidi, used purely as a scribe, does not satisfy the above criterion to require TGA regulation. The programme’s use, however, must be disclosed to the patient, and consent must be sought.

Locally, the government seems to have caught on to the above perceived advantages; the NSW Government’s Budget for 2026-2027 includes a near-AUD $40 million to introduce such scribing technology to “clinicians” as part of a “Future of Healthcare package” [21]. There are no further details as yet as to which “clinicians” will have access to such technology, what company (or companies) will provide it or how it will be regulated, although the Budget does allude to a “NSW Digital Assurance Framework” and “NSW AI Assessment Framework”, as well as the new establishment of an “AI Review Committee”.

The question of responsibility

One of the most surprising results of this survey to these authors was that nearly half of all respondents stated that, in adverse events, clinicians would and should bear sole responsibility for the use of AI software, and by corollary, companies developing this software would bear none. There is limited literature from Australia further exploring such questions, where studies focus mainly on specialist physicians and/or specific AI applications. Kovoor et al. [22] published one of the few studies, albeit a qualitative one, to focus on the adoption of artificial intelligence from a JMO perspective and were largely optimistic regarding the use of AI in future medical practice. They recommended that JMOs be the drivers of uptake of such technology and essentially act as liaison points between more senior doctors and regulatory bodies; little mention, however, is made of any prospective relationship of JMOs with AI software, or the companies that develop it, regarding responsibility when things go wrong.

The TGA explicitly regulates “software-based devices (including AI) that work to achieve a therapeutic purpose in human beings” [20]. The aforementioned exemptions to regulation appear to give clinicians some initial room to utilise AI so long as no therapeutic function is fulfilled; it is worth noting, however, that the Therapeutic Goods (Excluded Goods) Determination, which formally clears practitioners to use certain goods without the need for regulation by the TGA, dates from 2018, well before the most recent rapid developments in the sphere. They also explicitly comment on the risk of “scope creep”, utilising the example of a scribe that then is updated by its parent company to suggest diagnoses, thus meeting the definition of a software requiring regulation.

Once again, international perspectives may shed light on how Australia (and NSW) will proceed, and the challenges that will be faced. Donnelly [23] comments, for instance, on the South African situation and, rather than arguing primarily for stricter or clearer regulation of software or its developers, proposes a relaxation of the current restrictions to allow for greater uptake and development of new technologies. Ooi [24], in a more explicitly Australasian context, is quicker to place the onus strictly on regulators to take the lead, including that they invite developers of AI software to the table to flesh out proper regulation of the use of their technologies.

One partial solution is proposed again by Donnelly [23], who calls for an overhaul of the current South African system of medical liability, including advocating for compulsory insurance for doctors, should adverse events relating to AI technologies occur. It is a point of interest, assuming as it does two things: firstly, that AI will only proceed to be more permanently embedded in medical workflows and secondly, that, as he explicitly states in the South African context, a human agent as a registered health practitioner, i.e., doctor, will always be ultimately responsible for decisions made about patient care.

Part of the problem that Ooi [24] lights on in passing is that medical regulators do not necessarily have scope over the regulation of AI developers and companies, even in a strictly medical context. Recent developments regarding AI regulation more generally in the United States have provided a case in point, with widespread adoption of increasingly deregulated technologies outside of the hospital system; Wörsdörfer [25] cites ongoing concerns around not only inaccuracies in AI outputs but also the confidentiality of patient data, not only in the setting of data breaches but also in the setting of collapses or takeovers of companies that hold such data.

JMOs versus others

Precedents exist for research into the benefit AI may specifically provide to the workflow of JMOs. Using an AI scribe amongst Australian allied health professionals working in the private sector increased productivity by around 6% and decreased the time spent writing letters and documenting consultations [26]. These results would appear to show great promise for how a regulated AI scribe could impact JMOs working in the public sector.

In this study, the majority of JMOs agreed that they would feel comfortable having an AI scribe to summarise a patient interaction. This is likely due to the large amount of computer-based administrative work that usually falls to the JMO in the NSLHD. Almost all respondents stated that both current and future implementation of AI would be scribing and summarising patient interactions. They also agreed that one of the main advantages of AI would be freeing up the time spent on documentation for JMOs to perform other clinical tasks (and mitigate against needing to work overtime). This view is supported by findings from the United States, where one study of physicians, their assistants and nurse practitioners demonstrated an approximately 20% reduction in documentation from individual patient interactions and a reduction of 30% in the time spent working after hours when using an AI scribe [27].

With regard to investigations, the majority of JMOs in this study agreed that they would not be comfortable, at present, actioning a scan report or blood test summary produced solely by AI. Thematic analysis of answers revealed that participants wanted involvement from a doctor, who could be either themselves or a colleague, to feel more comfortable in actioning test results. Whilst some studies have reported that AI software has shown similar diagnostic ability to humans [28], the literature appears to overwhelmingly concur that human oversight is necessary from a diagnostic and ethical point of view [29]. Huang et al. [30] have posited that AI should be utilised in a reliable and efficient manner; future development should focus on how AI can complement the work that physicians already do, in order to reduce workload and improve efficiency.

One of the most scrutinised medical specialties with regard to the increasing use of AI is radiology. The theoretical advantage of using AI to provide initial scan analysis would reduce the workload of a radiologist and allow for a larger number of scans to be processed than currently; research is beginning to be published to confirm this supposition [31]. Whilst approximately one-third of JMOs identified AI-generated medical imaging reports as a current use, the majority of respondents felt it was more suitable as a potential future use. Thematic analysis of longer answers elicited concerns about the accuracy of AI-generated imaging reports and favoured the clinical experience of radiologists and their review of scans prior to actioning them.

The accuracy of AI-generated reports of medical imaging is mixed. A trial of technology to diagnose COVID-19 was reported to demonstrate an accuracy of 97.6% when analysing CT scans and chest X-rays, which improved the treatment of patients and reduced adverse outcomes [32]. A more recent study of AI-generated reports of CT and MRI scans of lung and breast nodules revealed that the technology’s accuracy matched or exceeded that of a radiologist [33]. However, other literature is not so supportive. One study of fractures on plain X-rays demonstrated worse performance by AI than radiologists [34], whilst a large meta-analysis conducted in 2025 by Takita et al. [35] found that AI had decreased accuracy in reporting imaging when compared to expert clinicians across a broad range of specialties.

Radiologists have exhibited concerns regarding the legal responsibility for scans reported by AI; specifically, concerns were reported over the lack of transparency in the programmes used for reporting [36]. Doctors of higher seniority than JMOs are generally more cautious in their attitudes, with previous findings of clinicians expressing fear of being replaced by AI, and that this will impact upon patient care [37]. A meta-analysis from Khalifa and Albadawy [38] recommended that for the most effective use of AI in medical imaging, increases in its diagnostic accuracy would be needed. Such concerns appear to support the view that AI is not yet suitably developed to produce adequately accurate medical imaging reports, even if it has the potential to do so [39]. The JMOs surveyed herein seem aware of this possibility and remain open to it.

Students and education

There have been studies involving medical students to evaluate their perceptions of AI, as well as studies of doctors; not only are they widely using the technology for their learning, but many also plan to use it in their early years of clinical work [40]. Evidence shows broad support and positive views towards AI nationally: medical students in Western Australia have shown interest in AI, even if they acknowledge that they do not understand its underlying principles or limitations [41]. Internationally, a study of Canadian medical students found that there was a high willingness to use and learn about AI whilst studying; respondents in that study, however, cited a discrepancy between willingness to learn about AI and available education programmes (whilst students in other degrees, such as engineering, received such learning opportunities) [42].

The JMOs surveyed in the NSLHD felt strongly that the use of AI should be taught in medical education, a view that seems to be supported by the fact that most JMOs rated their own knowledge of AI in healthcare as “average”. Its use amongst medical students is no longer theoretical outside of Australia: in one study from Pakistan, almost two-thirds of students use AI weekly, with the main advantages described as assistance with understanding difficult concepts and more precise answers to their clinical queries [43]. Many of these medical students felt that there was a reduction in the time needed to learn a concept when compared to more traditional learning methods. These very real, already widespread uses of AI further highlight the need for education to ensure not only that the use of AI is effective and ethical but also that there is an understanding and knowledge medical students can be equipped with regarding a technology likely to play an increasingly important part in their professional lives [44].

The question of public perception

In this study, over half of the JMO respondents felt that AI would impact their practice in a positive or very positive way. They did not demonstrate significant concern over any lack of transparency in the decision-making process when using AI, although it is worth noting that a recent survey of German doctors found that 75% of those surveyed identified it as a main concern [45]. JMOs in our survey also did not feel that public perception of the use of AI in clinical practice was a significant concern.

Studies of patient attitudes towards the use of AI by doctors have demonstrated change over time. Initial findings were of a positive response, although intuitively, a desire for human supervision of the technology was often voiced [46]. Later and more detailed research has revealed greater nuance in the views of patients; whilst some patient studies have found that they are in favour of the use of AI in the diagnosis of a pathology [47], broader international research has warned against blanket applications of technologies across patients in a single nation, or even a single region [48]. In other words, there is an increasingly strong subcurrent in research on patient attitudes towards AI that its blanket application may represent a further turn away from the patient-centred care movement.

How AI technologies can be tailored to ensure not only a safe, effective medical use but also a socially palatable use is a challenging question; the aforementioned difficulties of regulation only serve to muddy the waters. Ooi [24] provides perhaps the most practical view, explicitly positing that the expectations of patients, and society in general, need to be tempered regarding what AI can and cannot do, in a medical as well as more general context.

Limitations

There are some limitations to this study. For ease of data collection, the majority of questions were multiple choice or of a “yes/no/unsure” type. Data collection would benefit from longer-term, qualitative data collection methods such as longer free-text answers or interviews, to gain a better appreciation of the views of JMOs.

Involvement in this study was also voluntary and so may only have captured a certain population, with those holding stronger views on the subject of AI possibly more likely to respond. The views of a larger group of JMOs may represent less polarised opinions. All participants worked at one of two hospitals within a single local health district; these findings thus may not be replicated in the case of future larger-scale studies of other JMOs in the NSLHD, let alone in all of NSW or Australia. Further research across different sites and larger numbers of JMOs would be needed to draw more definitive conclusions.

Perhaps more importantly, it is worth noting also that the two hospitals amongst which JMOs this survey was distributed would be widely regarded as being well-staffed and well-resourced; these responses may not be replicated at less well-resourced hospitals, particularly those in rural Australia. Future research amongst this cohort of JMOs would be relevant, not just to assess the attitudes to AI but also to assess the feasibility of any rollout of technology, particularly in more remote areas.

## Conclusions

This study demonstrates that JMOs in the NSLHD have a varied understanding of AI both generally and in healthcare, although their attitudes towards it are broadly positive. Many use AI infrequently in their clinical work, but they do believe that it could reduce the burden of administrative tasks usually placed on JMOs, especially scribing interactions and summarising patient notes. They would, however, be more likely to use AI in both clinical and non-clinical work if it were endorsed by the local health district. JMOs expressed a desire to learn more about AI and agreed that it should have a place in medical education. Human oversight was felt to be needed, regardless of use and setting.

Future implementation of AI in the Australian public health system could alleviate the administrative burden on JMOs, although increased education and regulatory clarity, at the level of both local health districts and government, is required to bridge the gap between current and future use. The current lack of this clarity is a key barrier to broader AI uptake amongst JMOs, and international examples demonstrate not only the possible difficulty in providing such clarity but also the consequences of not doing so, given the pace of change both inside and outside of medicine. There exists clear potential for AI tools to reduce the administrative burden of the JMO; it is now the responsibility not only of them but also of healthcare administrators and government to make sure that such technology is implemented in a safe and responsible manner, with clarity surrounding processes when things go right and when things go wrong.

## Appendices

The survey used in the present study is shown in Table 4.

**Table 4:** Tabulated questionnaire questions AI: artificial intelligence

Question	Response
What is your age?	(Free text answer)
What is your current level of experience as a doctor?	<2 years; 2-5 years; 5-10 years; >10 years
Generally speaking, how would you categorise your views on AI?	Very positive; Positive; Neutral; Negative; Very negative
Please rate your knowledge of AI in general.	Very poor; Poor; Average; Good; Very good
Please rate your knowledge of AI in healthcare.	Very poor; Poor; Average; Good; Very good
On average, how often do you use AI in your clinical work?	Daily; Weekly; Fortnightly; Monthly; Never
How often do you use AI in your own personal education?	Daily; Weekly; Fortnightly; Monthly; Never
How often do you use AI when completing longer written work (e.g., research write-ups and reflections)?	Always; Frequently; Infrequently; Never
What is your overall opinion of how AI will impact your clinical practice?	Very positive; Positive; Neutral; Negative; Very negative
What do you think are the main advantages of AI? Please select at most four options.	Freeing time from documentation; Processing of large amounts of data; Assisting in clinical decision-making; Searching through data for best practice; Translation in real time; Better educated patients; Monitoring results, i.e., continuous monitoring and alerting of vital signs and monitoring of outpatient blood results; Triaging of patients in an outpatient/emergency department setting; Analysing of results; Other (free text)
What do you think are the current roles of AI? Please select at most four options.	Scribing notes; Summarising notes; Interpreting blood results; Interpreting scans; Drug synthesis; Surgery, e.g., assisting in robotic surgery; Clinical decision support; Personalised patient care; Patient education; Translation services in real time; Predicting disease trends; Predicting and managing pandemics; Medical education, e.g., simulation; AI has no role in clinical practice; Other (free text)
What are your main concerns in using AI at present? Please select at most four options.	The input of inaccurate information; The output of inaccurate information; Confidentiality issues; What my colleagues will think of me; What my patients/public will think of me; My own de-skilling; It may lead to over-reliance; Patients receiving inaccurate information; My responsibility if something goes wrong; Eradication of my job; Lack of transparency in decision making; Other (free text)
At present, would you be comfortable actioning a scan report done by AI?	Yes; No
If no, why not?	(Free text answer)
At present, would you be comfortable actioning a blood test summary made by AI?	Yes; No
If no, why not?	(Free text answer)
At present, would you be comfortable having AI scribe and summarise a patient consultation?	Yes; No
If no, why not?	(Free text answer)
What can you see as the future roles of AI?	Scribing notes; Summarising notes; Interpreting blood results; Interpreting scans; Drug synthesis; Surgery, e.g., assisting in robotic surgery; Clinical decision support; Personalised patient care; Patient education; Translation services in real time; Predicting disease trends; Predicting and managing pandemics; Medical education, e.g., simulation; AI has no role in clinical practice; Other (free text)
In an adverse medical event where AI was used, who holds the responsibility?	Clinician; Hospital/local health district; AI developer; Shared responsibility - clinician and hospital/local health district; Shared responsibility - clinician and AI developer; Shared responsibility - hospital/local health district and AI developer; Other (free text)
Would you be more likely to use AI programmes in your clinical or non-clinical work if they were endorsed by the LHD?	Yes; No; Unsure
As it stands, do you feel you would be open to incorporating AI into your clinical practice in the future?	Yes; No; Unsure
As it stands, do you feel you would be open to incorporating AI into other areas of your role in the future (e.g., education and research)?	Yes; No; Unsure
Should use of AI be taught in medical education?	Yes; No; Unsure
Do you think you should have to disclose the use of AI to your patients?	Yes; No
Where would you like to see AI implementation focused?	(Free text answer)
Please add any further thoughts you have about AI in medicine here.	(Free text answer)
